# Designing a Novel Functional Peptide With Dual Antimicrobial and Anti-inflammatory Activities *via in Silico* Methods

**DOI:** 10.3389/fimmu.2022.821070

**Published:** 2022-04-01

**Authors:** Min Kyoung Shin, Byungjo Lee, Seung Tae Kim, Jung Sun Yoo, Jung-Suk Sung

**Affiliations:** ^1^ Department of Life Science, Dongguk University-Seoul, Goyang, South Korea; ^2^ Life and Environment Research Institute, Konkuk University, Seoul, South Korea; ^3^ Animal Resources Division, National Institute of Biological Resources, Incheon, South Korea

**Keywords:** transcriptome, *in silico* analysis, antimicrobial peptide (AMP), anti-inflammatory peptide (AIP), TLR4 pathway

## Abstract

As spider venom is composed of various bioactive substances, it can be utilized as a platform for discovering future therapeutics. Host defense peptides are great candidates for developing novel antimicrobial agents due to their multifunctional properties. In this study, novel functional peptides were rationally designed to have dual antibacterial and anti-inflammatory activities with high cytocompatibility. Based on a template sequence from the transcriptome of spider *Agelena koreana*, a series of *via in silico* analysis were conducted, incorporating web-based machine learning tools along with the alteration of amino acid residues. Two peptides, Ak-N’ and Ak-N’m, were designed and were subjected to functional validation. The peptides inhibited gram-negative and gram-positive bacteria by disrupting the outer and bacterial cytoplasmic membrane. Moreover, the peptides down-regulated the expression of pro-inflammatory mediators, tumor necrosis factor-α, interleukin (IL)-1β, and IL6. Along with low cytotoxicity, Ak-N’m was shown to interact with macrophage surface receptors, inhibiting both Myeloid differentiation primary response 88-dependent and TIR-domain-containing adapter-inducing interferon-β-dependent pathways of Toll-like receptor 4 signaling on lipopolysaccharide-stimulated THP-1-derived macrophages. Here, we rationally designed functional peptides based on the suggested *in silico* strategy, demonstrating new insights for utilizing biological resources as well as developing therapeutic agents with enhanced properties.

## Introduction

Antibiotics have contributed to public health by reducing the risk of disease and infection since the first discovery of penicillin. However, antibiotic-resistant bacteria have become a serious health threat worldwide because of the misuse and overuse of antibiotics as well as the limitation of its application (1, 2). Following a primary infection, additional infections or failure of control on immune response can lead to fatal consequences, which are often accompanied by excessive or uncontrolled host immune defense. Therefore, there is an urgent demand for a new source of the multifunctional agent that not only compensates and substitutes the conventional drug but also enables systemic relief of the infection.

Host defense peptides (HDPs) are intrinsically expressed in a wide range of organisms, protecting against the pathogens as well as exhibiting immunomodulatory activities in innate immunity. The well-characterized group is antimicrobial peptide (AMP). The peptides possess pharmaceutical potency due to their high selectivity and efficacy towards biological components. Owing to their physiochemical nature, the utilization of peptides along with the optimization can aid drug repurposing and designing. Also, combined treatment of traditional medication and peptides has showed synergistic effects on various disease-related symptoms (3–5). The characterizing and identifying functional peptides are gaining biological values, expanding the utilization of the biological resources.

Functional compounds can be discovered by screening known chemicals or natural compounds from an existing source, and animal venom can be a great platform as it has evolved alongside host animals utilized for predation and defense (6, 7). Among venomous animal, spiders possess diverse venom components, as they are the most thriving terrestrial species. HDPs are often located in the venom and can protect the host from infections as well as serve several other functionalities, including anti-fungal properties, immunomodulation, wound healing, and cell differentiation (8–11). Despite their advantages, only a limited number of spider venom components have been studied so far, leaving thousands of HDPs to be identified.

High-throughput methods, including next-generation sequencing (NGS), are being actively utilized when processing and analyzing biological data and resources. Such technologies are effective when attaining transcriptome data of organisms that ate not widely known. (12). RNA sequencing and *de novo* assembly enable the construction of the transcripts without the genetic reference only using limited samples. Form the sequence-based homology prediction, information on structure and function can be generated from the transcriptome data with the incorporation of public databases (12, 13). In addition, functional components can be efficiently discovered with the aid of web-based tools before experimental verification. As the physiochemical properties can be calculated and the performance of machine learning and deep learning-based prediction tools have improved, it has become easier to discover biologically significant substances (14, 15).

In the present study, the transcriptome of *Agelena koreana* (*A. koreana*), a species of spider indigenous to Korea, was analyzed. The peptide sequence with potential antimicrobial function, c32159, was selected by performing a comparative analysis of homology and structural characteristics with known toxin peptides. Novel peptides were designed based on *in silico* analysis, including machine-learning prediction tools, structural modeling, and physiochemical property calculations. Two novel peptides Ak-N’ and Ak-N’m were selected and were tested for antibacterial activity against gram-negative and gram-positive bacteria, and their mechanism of action was also investigated. Additionally, the anti-inflammatory effect of the peptides was studied along with its underlying molecular pathway based on the immunomodulatory potential of the peptide. Our results suggested the discovery of novel peptides possessing dual property *via* rational design strategy as well as broadening the usefulness and utilization of transcripts of an organism.

## Materials and Methods

### Sample Preparation for RNA Sequencing

*A. koreana* spiders were collected from Jecheon, Chungcheongbuk-do, Korea. The spider venom glands were separated from the body and the total RNA extraction was conducted using TRIzol reagent (Life Technologies, Grand Island, NY, USA). RNA sequencing along with *de novo* assembly were performed by Macrogen (Seoul, Korea). Transcript profile from both the venom gland and the body was constructed in a transcriptome library. The transcriptome data generated for this study can be found in the NCBI’s Gene Expression Omnibus (GEO) and are accessible through GEO Series accession number GSE197102 (https://www.ncbi.nlm.nih.gov/geo/query/acc.cgi?acc=GSE197102). After designing the sequence, the peptides were synthesized by Biostem (Ansan, Gyeonggi-do, Korea) with purity above 95% and verified by mass spectroscopy and HPLC.

### Design of Functional Peptides by Machine Learning-Based Web Tools

Peptide c32159 was subjected to a 20-mer sliding window frame, followed by functional prediction using machine learning web-based tools. The anti-inflammatory property of the peptides were predicted using the servers AIPpred (http://www.thegleelab.org/AIPpred/) (16), PreAIP (http://kurata14.bio.kyutech.ac.jp/PreAIP/) (17), AntiInflam (http://metagenomics.iiserb.ac.in/antiinflam/) (18). For the prediction of the antimicrobial peptide, the following four models from servers a database of antimicrobial peptides (ADAM) (http://bioinformatics.cs.ntou.edu.tw/adam/) (19), AmpGram (http://biongram.biotech.uni.wroc P1/AmpGram/) (20), the support vector machine in CAMPR3 (http://www.camp3.bicnirrh.res.in) (21), and the the database of antimicrobial activity and structure of peptides (DBAASP) (http://dbaasp.org/) (22) were utilized. The hemolytic potential was obtained by the prediction from DBAASP against erythrocyte, hemolytic activity prediction for peptides employing neural networks (HAPPEN) (http://research.timmons.eu/happenn/) (23), and hemolytic peptide identification server (HemoPI) (http://crdd. Osdd.net/aghava/hemopi/) (24).

### Bacterial Strains and Cell Lines

Every strain used in the study was purchased from the Korean Culture Center of Microorganisms (KCCM, Seoul, Korea) or the American Type Culture Collection (ATCC, Manassas, VA, USA). The following bacterial strains were used in this study: *Escherichia coli* KCCM 11234 (*E*. *coli*), *Pseudomonas aeruginosa* ATCC 9027 (*P*. *aeruginosa*), *Bacillus cereus* KCCM 21366 (*B*. *cereus*), *Staphylococcus aureus* KCCM 11335 (*S*. *aureus*), and methicillin-resistant *S*. *aureus* ATCC 33591 (MRSA). Bacterial strains were maintained in tryptic soy broth (TSB, Difco Laboratories, Detroit, MI, USA) and cultured under shaking incubation at 37°C. In addition, the following human cell lines were used in this study: THP-1 monocytes (ATCC TIB-202), primary adipose-derived mesenchymal stem cell (hADMSC; CEFO Co., Seoul, Korea), normal bronchial epithelial cells BEAS-2B (ATCC CRL-9609), and lung carcinoma cells H460 (ATCC HTB-177). THP-1 and H460 cells were cultured in RPMI 1640 medium (Welgene Inc., Gyeongsan, Gyeongsangbuk-do, Korea) supplemented with 10% fetal bovine serum (Gibco), 1% penicillin and streptomycin (Gibco), and sodium pyruvate (Welgene Inc.). hADMSC and BEAS-2B cells were cultured in CEFOgro™ Human MSC Growth Medium (CEFO Co.) and BEGM™ Bronchial Epithelial Cell Growth Medium BulletKit™ (Lonza, Basel, Switzerland), respectively. The cells were maintained under humidified air with 5% CO_2_ at 37°C.

### Antibacterial Activity Assay and Minimum Inhibitory Concentration Determination

Two-fold broth microdilution assays were performed to measure the MIC values of the peptides. In brief, the strains were grown to an exponential phase in MHB at 37°C and then diluted to reach 2×10^5^ CFU/mL in fresh media. Peptides were serially diluted in PBS and then added of 50 μL/well to a 96-well plate. An equal volume of bacterial suspensions was added to each well, resulting in final concentrations of peptides ranging from 1 to 32 μM. The wells only with the medium served as blank, while adding the medium to inoculant was used as control. The plates were incubated at 37°C for 18 h, and the bacterial growth was measured at 600 nm using a microplate reader (Molecular Devices, Sunnyvale, CA, USA). MIC values were determined as the lowest concentration of peptides without observed bacterial growth comparable to blank. To perform colony forming assay, different concentrations of peptides were mixed with an equal volume of bacterial suspensions, resulting in 2×10^5^ CFU/mL. The samples were spread onto tryptic soy agar (TSA, Difco Laboratories) plates after 3 h incubation and incubated overnight at 37°C. Relative colony formation was measured by counting colonies formed on agar plates, and was compared with the control plate for the relative expression.

### Membrane Permeabilization Measurement

To assess the outer membrane permeation by the peptides, 1-N-phenylnaphthylamine (NPN, Sigma-Aldrich, St. Louis, MO, USA) uptake assay was conducted. After washing with 5 mM 4-(2-Hydroxyethyl)piperazine-1-ethanesulfonic acid (HEPES, Sigma-Aldrich) buffer three times, the Gram-negative bacteria was prepared in the same buffer at a concentration of 1×10^8^ CFU/mL. The 100 μL of suspension was transferred to a black 96-well microplate and then added with 50 μL of 40 μM NPN. The fluorescence was measured for 15 min immediately after 50 μL peptides of 4 × MIC were added (excitation: 350 nm; emission: 420 nm). 3, 3’-dipropylthiadicarbocyanine iodide (DISC_3_(5), Sigma-Aldrich) was used to measure depolarization of the cytoplasmic membrane by the peptides. The bacteria were prepared for 10 mL of 1×10^7^ CFU/mL in 5mM HEPES buffer containing 20mM glucose (Sigma-Aldrich) and 0.1 M KCl (Sigma-Aldrich). After washing three times, bacterial cells were resuspended in the buffer with the final concentration of 0.4 μM DISC_3_(5), and 100 μL of suspensions were added to a black 96-well microplate. The plate was incubated for 30 min at 37°C in the dark, followed by fluorescence measurement (excitation: 622 nm; emission: 670 nm). The fluorescence was recorded for 10 min immediately after 100 μL peptides of 2 × MIC were dispensed to each well. The fluorescent signal was read by Infinite F200 Pro multimode microplate reader (Tecan, Männedorf, Switzerland) during every assay.

### Field Emission Scanning Electron Microscope Imaging

Mid-log phase *E. coli*, *S. aureus*, MRSA were seeded on cover slides coated with poly-L-lysine (Sigma-Aldrich). Each sample was treated with 1 × MIC of peptides for 2 h and was fixed overnight using 2.5% glutaraldehyde (Sigma-Aldrich). The samples were sequentially dehydrated by 30, 50, 60, 70, 80, 90, and 100% ethanol, coated with gold, and imaged *via* Hitachi S-4300 FE-SEM (Tokyo, Japan).

### Cell Viability Assay

THP-1, hADMSC, BEAS-2B, and H460 cells were seeded in 96-well plates at a density of 1×10^5^ cells/mL to evaluate the cytotoxic effect of the peptides. Different concentrations of peptide were treated and incubated for 24 h. Quanti-Max WST-8 Cell Viability assay solution (Biomax, Seoul, Korea) was added to each well, and then the plate was incubated for 1 h. The absorbance was read at 450 nm using a microplate reader (Molecular Devices), and the relative cell viability was calculated.

### Hemolytic activity

The hemolytic activity of the peptide was tested using bovine red blood cells (RBCs) purchased from Innovative Research (Novi, MI, USA). RBCs were diluted in PBS to obtain a 4% (v/v) suspension and were aliquoted at 100 μL per microtube. The RBC suspensions were mixed with an equal volume of 2-fold peptides and incubated at 37°C for 1 h. PBS and 0.1% Triton X-100 served as the negative control (0% hemolysis) and positive control (100% hemolysis), respectively. The tubes were centrifuged at 3,000 × *g* for 10 min at 4°C, and the supernatants were transferred to a 96-well microplate. The absorbance (Ab) was measured at 450 nm using a microplate reader (Molecular Devices). The hemolytic activity of each peptide was calculated by (Ab_peptide_ – Ab_PBS_)/(Ab_Triton X-100_ – Ab_PBS_) × 100.

### Serum Stability Test

Peptide stability in human serum (HS, Sigma-Aldrich) was tested by sodium dodecyl sulfate-polyacrylamide gel electrophoresis (SDS-PAGE) (25). 25 μg of peptides were incubated with 2%, 5%, and 10% of HS in a total volume of 20μL. The samples, peptide control, HS controls were incubated at 37°C for 1, 4, 12 h and were mixed with 5 × protein sample buffer. The samples were loaded in 12% SDS gel along with Prosi prestained protein marker (GenDEPOT, Katy, TX, USA). After separation, gels were stained with Coomassie Brilliant Blue R-250 staining solution (Bio-Rad, Hercules, CA, USA). Gels were destained and imaged by ChemiDoc™ Imaging Systems (Bio-Rad).

### Enzyme-Linked Immunosorbent Assay and Reverse Transcription Quantitative Polymerase Chain Reaction

THP-1 cells were differentiated into macrophages by treatment of 50 ng/mL phorbol-12-myristate-13-acetate (PMA, Sigma-Aldrich) for 24 h. Cells were treated with peptides with or without lipopolysaccharide (LPS, Sigma-Aldrich) for 24 h. Supernatants were collected to determine secreted tumor necrosis factor-α (TNF-α), interleukin (IL)-1β, and IL-6 protein levels using Quantikine ELISA kits (R&D Systems, Minneapolis, MN, USA) according to the manufacturer’s instructions. Total RNA was isolated from the remaining cells and 2000 ng of RNA was reverse transcribed into cDNA by M-MLV Reverse transcriptase (ELPISBIO, Daejeon, Korea). RT-qPCR was performed by using SYBR Green PCR master mix (KAPA Biosystems, Wilmington, MA, USA) and the synthesized cDNA. The cDNA was amplified using primer sets for TNF-α, IL-1β, IL6, interferon alpha-2 (IFNA2) and beta-1 (IFNB1), where GAPDH served as an internal control (**Supplementary Table 1**). The following condition was used: denaturation of template at 95°C for 1 min, 40 cycles of denaturation at 95°C for 20 s, annealing at 60°C for 20 s, and extension at 72°C for 20 s. The relative fluorescence was analyzed using CFX Connect™ Real-Time PCR Detection System (Bio-Rad, Hercules, CA, USA).

### Western Blot Analysis

Cells were washed and lysed using RIPA buffer (Biosolution, Seoul, Korea) containing protease and phosphatase inhibitor cocktails (Sigma-Aldrich). After protein quantification using Pierce™ BCA Protein Assay Kit (Thermo Fisher Scientific, Waltham, MA, USA), an equal amount of protein from each sample were separated by 10% SDS-PAGE. Proteins were transferred onto polyvinylidene difluoride membranes, which were blocked using 5% skim milk (Difco Laboratories). Primary antibodies (1:1000 dilution) were incubated at 4°C overnight, and secondary antibody conjugated with horseradish peroxidase (1:2000 dilution) was applied at room temperature for 45 min after washing. ECL Plus Western blotting detection reagents (Amersham Bioscience, Buckinghamshire, UK) was used for protein detection and was imaged by ChemiDoc™ Imaging Systems. Acquired data were quantified by Image Lab™ Software (Bio-Rad).

### Immunocytochemistry Analysis

After seeding on cover slides, the differentiated THP-1 cells were stimulated by LPS with or without peptide (2 μM) for 2 h. Cells were washed with ice-cold phosphate buffered saline (PBS, Gibco), fixed with 4% formaldehyde (Sigma-Aldrich), and permeabilized with 0.25% Triton X-100 (Sigma-Aldrich). Samples were blocked with 2% bovine serum albumin (BSA, Santa Cruz Biotechnology Inc., Dallas, Texas, USA) in PBS for 1 h at room temperature and incubated with p-p65 antibody (p-NF-κB, 1:500, Santa Cruz Biotechnology Inc.) overnight at 4°C. Following washing, the cells were incubated with anti-mouse Alexa Flour 555 antibody (1:1000, Cell Signaling Technology, Danvers, MA, USA) for 1h at room temperature and stained with 1 μg/mL 4′,6-diamidino-2-phenylindole (DAPI, Thermo Fisher Scientific). The prepared samples were visualized under Leica TCS STED CW microscope (Leica-Microsystems, Mannheim, Germany).

### Flow Cytometry Analysis

THP-1-derived macrophages were stimulated by LPS with or without the peptide for 30 min in a humidified incubator at 37°C. The cells were separated from the dish using cell dissociation buffer (Gibco) and incubated with Toll-like receptor 4 (TLR4) antibody (1:100, Santa Cruz Biotechnology Inc.) on ice for 1 h. Following washing, samples were incubated with anti-mouse Alexa Flour 488 antibody (1:1000, Cell Signaling Technology) on ice for 45 min. Cells were resuspended in analysis buffer (1% BSA and 0.01% sodium azide in PBS) and analyzed for surface TLR4 by BD LSRFortessaTM Flow Cytometer (BD Biosciences, San Jose, CA, USA).

### Statistical Analysis

All experiments were conducted in triplicate and the results were expressed as mean ± standard error of mean (SEM). The statistical significance of the data was evaluated by performing a one-way ANOVA test followed by Tukey’s post-test using GraphPad Prism 9.3.1 (GraphPad Software, La Jolla, CA, USA). *P*-values of <0.05 were considered statistically significant.

## Results

### Transcriptome Analysis of the Spider *A. koreana* and the Identification of the Template Sequence c32159 Transcript

**Figure 1:**
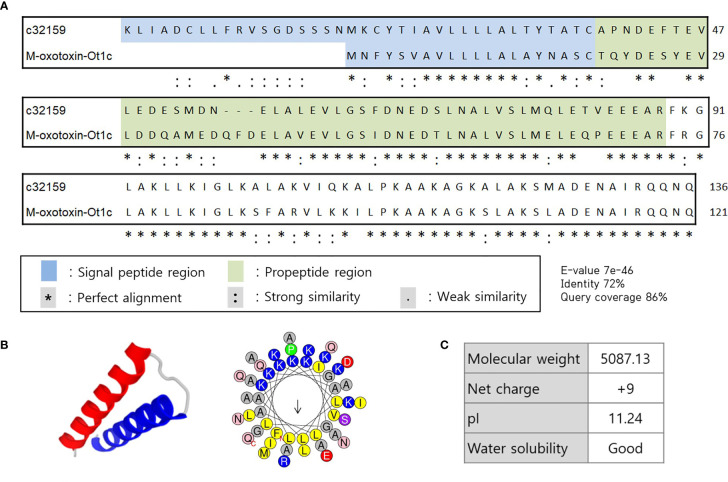
Sequence alignment and structural representation of c32159. **(A)** High homology was revealed by BLAST on signal peptide region, propeptide region, and mature peptide of c32159 transcript. “*”, “:”, “.” indicates perfect alignment, strong similarity, and weak similarity, respectively. **(B)** *In silico* analysis predicted mature sequence of c32159 to have helix-loop-helix structure with distinct hydrophilic and hydrophobic faces. **(C)** Physiological properties were calculated of the sequence.

In search of HDP from the spider transcriptome, *A. koreana* was subjected to RNA sequencing using the NGS technique and proceeded for the RNA library construction. A total of 108,412,884 reads were sequenced from the sample, and the following *de novo* assembly resulted in 150,997 transcripts. The RNA library was constructed with the GC content and the N50 length of 34.83% and 1,182 bpm, respectively. The transcripts expressed in the venom gland were analyzed with the Basic Local Alignment Search Tool (BLAST) against the known sequences in the NCBI database for the screening of transcripts above 60% identity and/or 60% DB coverage. Since toxin peptides are generally cleaved off of signal and propeptide regions to functionally mature, SignlaP and SpiderP programs were used. Amongst them, c32159 showed high homology with M-oxotoxin-Ot1c from the spider venom of *Oxyopes takobius* (26). c32159 consisted of a lengthy 48-mer mature peptide with a 38-mer signal sequence and a 50-mer propeptide region. As it was suggested that c32159 is a spider toxin peptide, the structural and physiological analysis was conducted. The sequence was predicted to be a strong AMP, considering its α-helical structure and amphiphilic property with a high net charge of +9, but simultaneously thought to show high cytolytic activity (**Figure 1**) (27, 28). Thus, c32159 was selected for the template sequence for designing functional peptides with additional anti-inflammatory property and enhanced biological applicability.

### Design and Characterization of Functional Peptides *via In Silico* Methods

In order to design peptides with dual functionality with low cytotoxicity, a series of *in silico* analysis was conducted according to a rational design strategy. First, the c32159 sequence was subjected to a sliding window of 20-mer sequences, producing 29 analogs with 20 amino acid residues. For each generated sequence, independent machine learning-based web tools were used to predict antimicrobial, anti-inflammatory, and hemolytic activity (**Supplementary Table 2**). The general scoring of the prediction results was used for the selection of the five most powerful candidates. Following was the calculation of physiological properties of sequences, including molecular weight, net charge, pI, water solubility, secondary structure, and α-helix propensity (**Supplementary Table 3**). Then, five sequences were mutated for each single amino acid to generate a list of mutated sequences. Based on the analysis, new sequences that retain the AMP property were again submitted to the workflow of prediction tools.

Finally, a pair of sequence was secured; a sequence that was predicted to have the most functionality among the template-origin sequences and another sequence that was expected of improved functionality through the mutation from that previous sequence. The 20-mer of the N terminal of the original peptide, c32159, was selected and named Ak-N’. The mutated form of Ak-N’, in which three residues were mutated, was named Ak-N’m. The prediction score of Ak-N’m by machine learning-based tools improved in the anti-inflammatory peptide (AIP) efficacy, while it was reduced in hemolytic activity (**Table 1**). The net charge was lowered from +5 to +3, and the hydrophobic face was calculated to be narrower (**Table 2**). So far, we devised a method to improve the functionality of the peptide obtained from the spider transcript, and functional validation and assessment was conducted with two peptides.

**Table 1:** Functional prediction of Ak-N’ and Ak-N’m using machine learning-based tools.

Sequence	AIP prediction			AMP prediction				Hemolysis prediction		
	AIPpred	PreAIP	AntiInflam	ADAM	AmpGram	CAMPR3 (SVM)	DBAASP	DBAASP (erythrocyte)	HAPPEN	HemoPI
Ak-N’ (FKGLAKLLKIGLKALAKVIQ)	0.5558	0.618	0.893109	2.56	1	0.960	AMP	Active	0.489	0.50
Ak-N’m (NKGLAKLLKIGLKALESVIQ)	0.6273	0.627	2.39178	1.89	0.9963	0.897	AMP	Not active	0.063	0.42

**Table 2:** Physiochemical properties of Ak-N’ and Ak-N’m .

	Ak-N’	Ak-N’m
Sequence	FKGLAKLLKIGLKALAKVIQ	**N**KGLAKLLKIGLKAL**ES**VIQ
Net charge	+5	+3
Mw	2152.75	2136.62
pI	11.28	10.68
Water solubility	Good	Good
Hydrophobic face	IGALVGLLLFI	IGALVGLLL
Helical structure	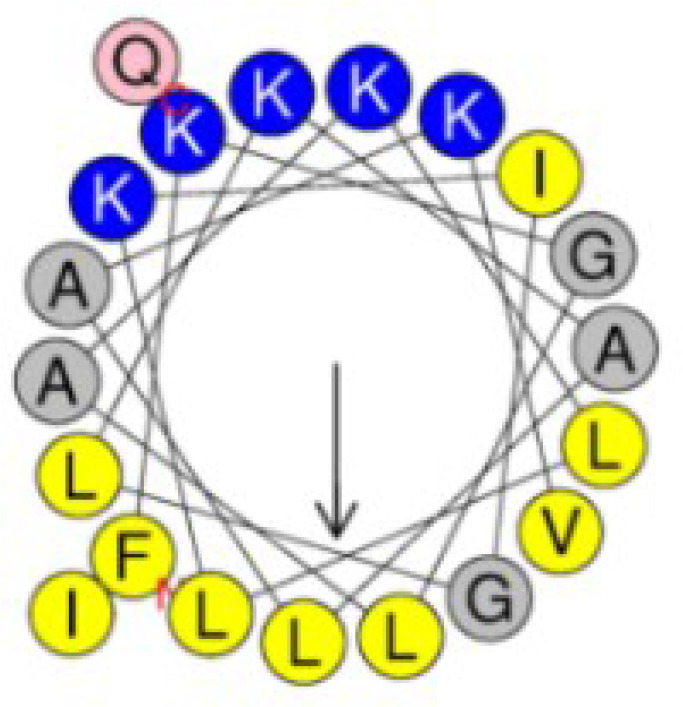	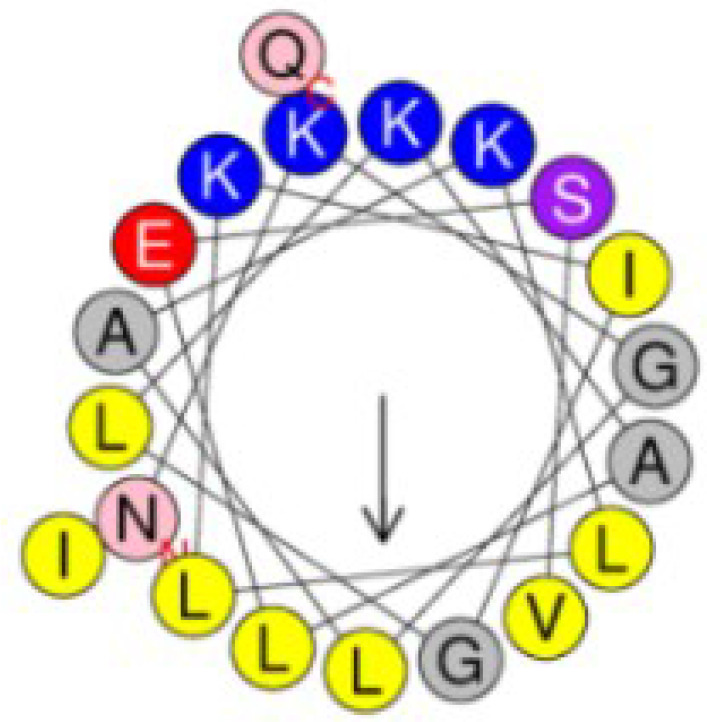

### Antibacterial Effects of Ak-N’ and Ak-N’m on Gram-Negative and Gram-Positive Bacteria

**Figure 2:**
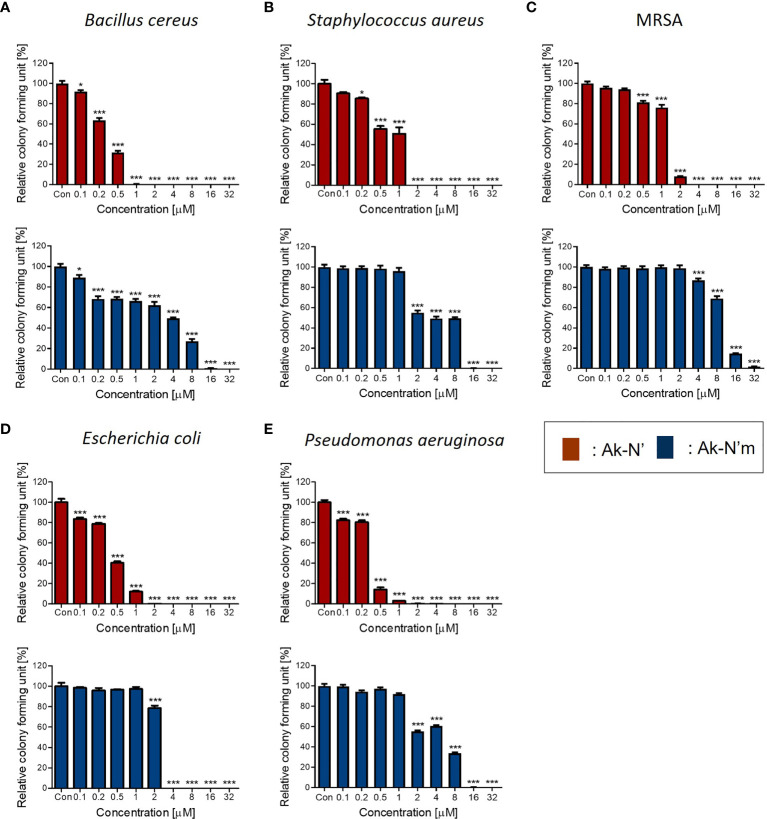
Antibacterial activity of the peptides Ak-N’ and Ak-N’m. Colony forming assay was conducted to validate the antimicrobial effects of the peptides ranging 0.1 μM to 32 μM. Inhibitory effects of Ak-N’ (red) and Ak-N’m (blue) on **(A)** *E*. *coli*, **(B)** *P*. *aeruginosa*, **(C)** *B*. *cereus*, **(D)** *S*. *aureus*, and **(E)** MRSA were shown. Data are presented as mean ± SEM. *p < 0.05, ***p < 0.001 compared to the control.

The antibacterial activity of the peptides was evaluated by performing the CFA and MIC determination to verify the prior prediction results. Common types of pathogenic bacteria that cause infectious diseases were selected. The peptide concentrations of 0.1 to 32 μM were tested against bacterial strains for the CFA (**Figure 2**). In the case of Ak-N’, treatment of the peptide resulted in complete inhibition of growth on all strains within a concentration of 8 μM. Ak-N’m showed significant antibacterial activity against all of the strains in a dose-dependent manner. MIC values were determined by treating various concentrations (0.1 ~ 128 μM) of the peptides for 24 h (**Table 3**, **Supplementary Figure 1**), of which all of the values were below 32 μM. Bacterial strains were more susceptible to Ak-N’, suggesting that Ak-N’ is a stronger AMP than Ak-N’m as expected. The overall results demonstrated the strong inhibitory effect of Ak-N’ and Ak-N’m on both gram-negative and gram-positive bacteria, and the mechanism of action was investigated accordingly.

**Table 3:** MIC values of Ak-N’ and Ak-N’m.

MIC (μM)	*E. coli*	*P. aeruginosa*	*B. cereus*	*S. aureus*	MRSA
Ak-N’	2	2	4	0.2	2
Ak-N’m	2	8	32	8	32

### Bacterial Membrane Disruption by Ak-N’ and Ak-N’m

The interaction of cationic AMPs with cellular membrane components is known to be one of the mechanisms for bacterial cell death. When a sufficient amount of AMPs accumulates on bacteria, it leads to pore formation and membrane disruption, causing the rapid killing of cells. A peptide with high antibacterial activity from the honey bee venom, melittin, was used as a positive control. The concentrations of 1 × MIC for each peptide were selected for the following assays.

**Figure 3:**
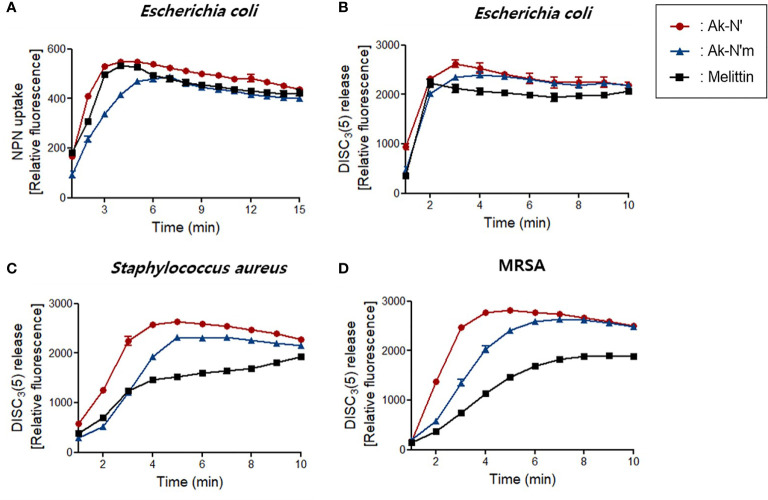
Bacterial membrane permeation by Ak-N’ and Ak-N’m. **(A)** NPN uptake showed disruption of the outer membrane by 1 × MIC of Ak-N’ and Ak-N’m on *E*. *coli*. **(B-D)** Depolarization of bacterial cytoplasmic membrane was measured using DISC_3_(5) dye. The peptides induced rapid increase of fluorescence in *E. coli*, *S. aureus*, and MRSA within 5 min, showing comparable or stronger disruption of the bacterial membrane than melittin.

First, the NPN uptake assay was performed to measure the permeabilization of the outer membrane of gram-negative strains. NPN fluoresces strong signals when it is introduced into hydrophobic conditions, in this case, entering the inner portion of the membrane by pore formation. When fluorescent was recorded for 15 min after peptide treatment, a rapid increase in fluorescence signal was observed in both Ak-N’ and Ak-N’m treatment, which was similar or higher than that of melittin (**Figure 3A**).

The fluorescent dye DISC_3_(5) was used to assess the destruction of the bacterial cytosolic membrane, which fluoresces fluorescent when leaked out of cells by membrane depolarization. DISC_3_(5) was stabilized into the cytoplasm before the addition of the peptide. Likewise, the treatment of the peptides induced an instant spike of signal in all of the bacterial strains. Gram-positive bacteria were more vulnerable to the peptides as the fluorescence intensity was stronger than that of the treatment of melittin (**Figures 3B-D**). The overall results indicated that Ak-N’ and Ak-N’m are AMPs with a strong antibacterial activity that can permeabilize both the outer membrane and the cytosolic membrane of bacteria.

**Figure 4:**
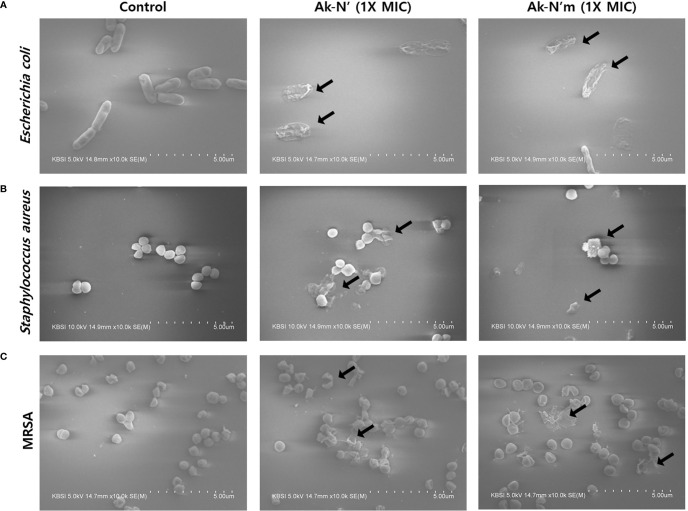
Morphological changes of bacteria caused by Ak-N’ and Ak-N’m. Effects on bacterial morphology upon peptide treatment was observed by FE-SEM imaging. When treated with 1 × MIC peptide for 2 h, **(A)** *E. coli*, **(B)** *S*. *aureus*, and **(C)** MRSA all showed damage to bacterial membranes with blebs and ruptures compared with the negative control treated with an equal volume of PBS.

The morphology of bacterial cells was observed by FE-SEM imaging to confirm the bactericidal activity of AMPs Ak-N’ and Ak-N’m. *E. coli*, *S. aureus*, and MRSA were treated with 1 × MIC of each peptide for 2 h and then compared with the control group treated with PBS. As shown in **Figure 4**, the controls showed a normal structure with an intact membrane. In contrast, the cells exposed to the peptides exhibited deformed bacterial surfaces with loss of integrity and even complete disruption of the cell.

To sum up, antibacterial assays revealed that Ak-N’ and Ak-N’m are novel AMPs with high antibacterial activity as intentionally designed. While both peptides effectively destroyed bacterial membrane and structure, Ak-N’ was proven to be a stronger AMP in agreement with the previous prediction, supporting *in silico* analytical processes and its results.

### Evaluation of Cytotoxicity and Hemolytic Activity of Ak-N’ and Ak-N’m

**Figure 5:**
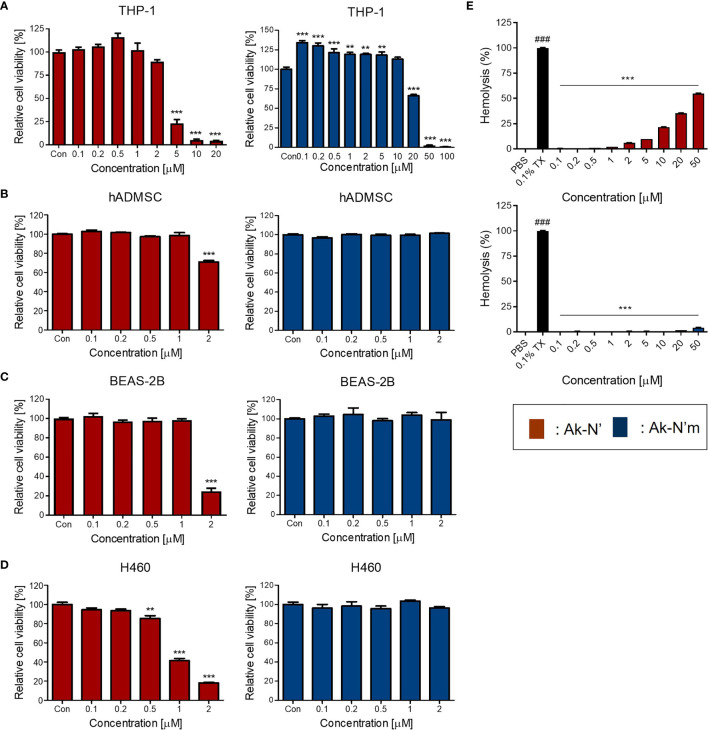
Evaluation of cytotoxicity of the peptides on human cell lines and bovine red blood cells. Cell viability assay was conducted to evaluate the cytotoxic effect of peptides Ak-N’ (red) and Ak-N’m (blue) using various concentrations. **(A)** THP-1, **(B)** hADMSC, **(C)** BEAS-2B, and **(D)** H460 cells were seeded at a density of 1×10^4^ cells/well and WST-8 solution was used to measure the cell viability. **(E)** Hemolytic activity was evaluated using bovine RBCs by treating peptide ranging from 0.1 to 50 μM. Ak-N’ showed hemolysis in a dose-dependent manner exceeding 50%, while Ak-N’m disrupted less than 5% of RBCs. Data are presented as mean ± SEM. **p < 0.01, and ***p < 0.001 compared to the control. ^###^p < 0.001 compared between the negative control (PBS) and the positive control (0.1% Triton X-100).

Prior to investigating the anti-inflammatory efficacy, cell viability and hemolysis assay were conducted to confirm the intended reduction of cytotoxicity of the peptides. A human monocyte cell line THP-1, an *in vitro* inflammation model, as well as hADMSC, BEAS-2B, and H460 cells were treated with different concentrations of each peptide. The cell viability assay results showed that the viability of THP-1 and the rest of the cell lines was not affected by Ak-N’m treatment up to 10 μM and 2 μM, respectively. In the case of Ak-N’, it exhibited a significant cytotoxic effect toward THP-1 from a concentration of 5 μM and on primary and normal cell lines from 2 μM. Interestingly, the cell viability of H460 was significantly affected by Ak-N’ from a concentration of 0.5 μM, which was lower than that of other cell lines, suggesting the possibility of anti-cancer activity of the peptide (**Figures 5A–D**).

The hemolytic activities of the peptides were evaluated using bovine erythrocytes. Hemolytic activity was presented as the relative value to 0.1% Triton-X treatment, exhibiting 100% hemolysis, and the concentrations between 0.1 and 50 μM of Ak-N’ and Ak-N’m were tested. When treated up to 50 μM, Ak-N’m caused almost no hemolysis which was below 5%, whereas Ak-N’ caused hemolysis over 50% in a dose dependent manner (**Figure 5E**).

Based on the results, it was confirmed that the modification of Ak-N’ into Ak-N’m by triple amino acid mutation resulted in a significant reduction of cytotoxicity to suit the intended goal of the peptide design. Peptides were additionally tested for stability in the presence of HS, where both peptides exhibited stability after incubating with 10% HS at 37°C for 12 h (**Supplementary Figure 2**). Subsequently, the concentration of 0.1 to 2 μM for Ak-N’ and 0.1 to 10 μM for Ak-N’m was selected to investigate the anti-inflammatory effects of the peptides, taking into account the concentration range in which each peptide exhibits cytotoxicity on THP-1 cells.

### Anti-Inflammatory Effects of Ak-N’ and Ak-N’m on Macrophage

The anti-inflammatory activity and its mechanism were studied using M0 macrophage and M1 macrophage that were activated by LPS. The peptides were co-treated with LPS on M0 cells for 24h to investigate their functionalities. Major pro-inflammatory cytokines, TNF-α, IL-1β, and IL-6, were selected and evaluated for their expression among experimental groups. The changes in mRNA expression and protein levels of the cytokines were assessed *via* qRT-PCR and ELISA, respectively.

**Figure 6:**
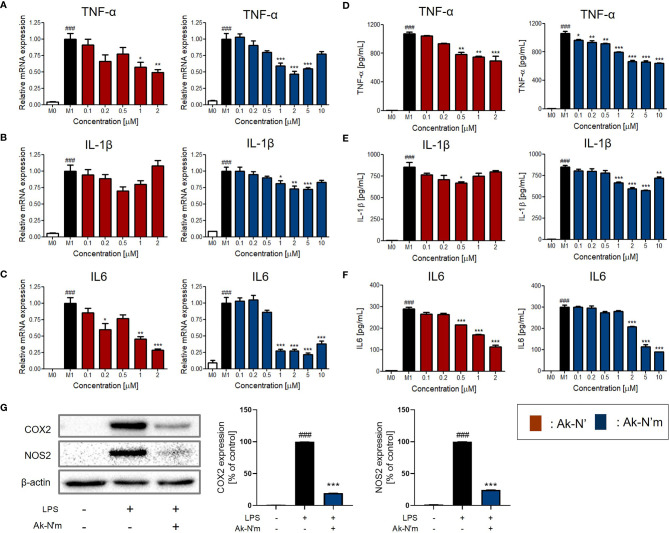
Inhibition of pro-inflammatory mediators by Ak-N’ and Ak-N’m on LPS-stimulated macrophages. Treatment of Ak-N’ (red) and Ak-N’m (blue) was evaluated of the changes on mRNA expression level. **(A)** TNF-α, **(B)** IL-1β, and **(C)** IL6 were measured by qRT-PCR upon co-treatment of LPS (1 μg/mL) and the peptide. The secreted cytokine levels in the cell supernatant were measured by ELISA after treatment the peptide with or without LPS. The levels of **(D)** TNF-α, **(E)** IL-1β, and **(F)** IL6 decreased when peptides were co-treated compared to the single treatment of LPS. **(G)** The protein level of COX2 and NOS2 decreased when simultaneously treated with LPS and 2 μM Ak-N’m for 24h. Data are presented as mean ± SEM. ^###^p < 0.001 compared to the control group; *p < 0.05, **p < 0.01, and ***p < 0.001 compared to the LPS-stimulated group.

The results indicated that the production of pro-inflammatory mediators was significantly inhibited by Ak-N’ and Ak-N’m, proving their efficacy as AIPs (**Figures 6A–F**). Treatment of Ak-N’ showed a dose-dependent decrease in mRNA expression except for IL-1β. The relative mRNA level of TNF-α and IL-6 was the lowest in 2 μM, each decreased by 0.5- and 0.25-fold. Ak-N’m exhibited stronger anti-inflammatory effects with a broader concentration range, among which IL6 was the most inhibited cytokine.

Considering the previous results altogether, it was validated that the design of the functional peptide and its workflow were effective and rational. Notably, Ak-N’m was presented as a functional peptide with significant dual functionality serving the purpose of our study. In addition, as Ak-N’m suppressed the protein level of cyclooxygenase 2 (COX2) and nitric oxide synthase 2 (NOS2) that are key players in the inflammatory signaling pathway, the functional mechanism of the peptide was further investigated (**Figure 6G**).

### Interaction Among TLR4, CD14 and Ak-N’m Related to Anti-Inflammatory Property

To investigate the underlying mechanism of Ak-N’m anti-inflammatory property, the LPS-induced TLR4 signaling pathway of THP-1 was targeted. The pathway is initiated by the binding of LPS to TLR4, which is followed by TLR4 dimerization. Therefore, the association between Ak-N’m and the macrophage surface receptors was studied. M0 macrophages were blocked with anti-TLR4 and anti-CD14 antibodies or treated with the peptide and then stimulated with LPS to be observed the changes in pro-inflammatory cytokine expression.

**Figure 7:**
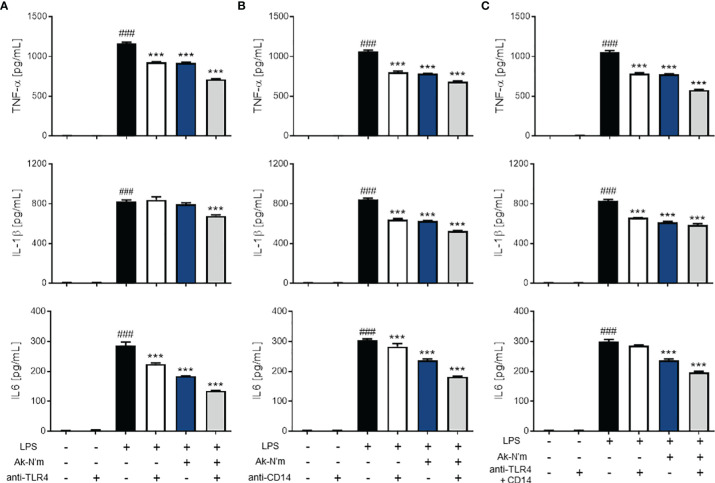
Anti-inflammatory effects of Ak-N’m by interaction with TLR4 and CD14. To evaluate the interaction capability of the peptide with macrophage surface receptors, Ak-N’m (2 μM) was treated and compared with the functional inhibition of the receptors by their antibodies. **(A)** TLR4 blocking, **(B)** CD14 blocking, and **(C)** simultaneous blocking of TLR4 and CD14 was conducted *via* antibody treatment (5 μg/mL). The inhibition of cytokine secretion (TNF-α, IL-1β, IL6) was compared among LPS-stimulated (black), antibody-treated (white), Ak-N’m-treated (blue) and antibody/Ak-N’m-co-treated (gray) groups. Treatment of Ak-N’m showed similar or stronger inhibitory effect on macrophage surface receptor than a single antibody treatment. ^###^p <0.001 compared difference between the negative and the positive group. Data are presented as mean ± SEM. ***p <0.001 indicates significant difference against LPS-stimulation with antibody and/or Ak-N’m treatment.

Anti-TLR4 and anti-CD14 antibodies were able to inhibit inflammatory responses *via* recognizing the active sites of their antigens. The antibody-treated groups (TLR4 single blockade, CD14 single blockade, and co-blockade) were compared with the peptide-treated groups. The treatment of Ak-N’m exhibited a similar or stronger inhibitory response on all three cytokines, TNF-α, IL-1β, and IL-6, than that of the suppression of inflammatory response by antibodies (**Figure 7**). This suggested that the peptide may act as an inhibitor that interacts with receptors related to the TLR4 pathway comparable to the functional blocking by an antibody. In addition, it was proposed that Ak-N’m targets more than one protein since the co-treatment of the peptide and antibody exhibited synergistic inhibition on inflammation. Accordingly, the effect of Ak-N’m on TLR4 downstream, Myeloid differentiation primary response 88 (MyD88)-dependent pathway and TIR-domain-containing adapter-inducing interferon-β (TRIF)-dependent pathway, was investigated.

### Inactivation of MAPK Signaling Pathway by Ak-N’m

**Figure 8:**
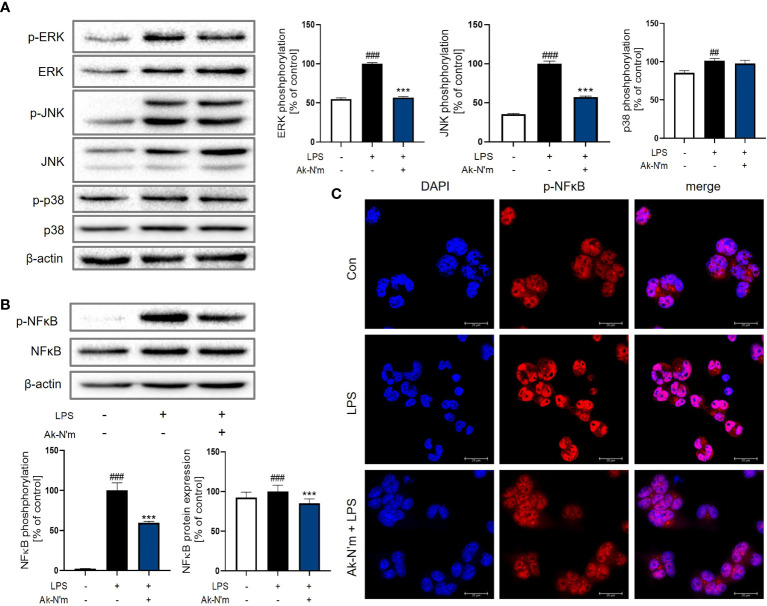
Inhibition of MAPK pathway by Ak-N’m in macrophages. Ak-N’m affected MyD88-dependent pathway by inhibiting MAPK signaling. **(A)** Western blot analysis showed inactivation of ERK and JNK by 2 μM Ak-N’m treatment. **(B)** The expression and activation of the transcription factor NF-κB was suppressed by the peptide. **(C)** The nuclear translocation of NF-κB was inhibited by AK-N’m on LPS-stimulated macrophages. Data are presented as mean ± SEM. ^##^p < 0.01 and ^###^p < 0.001 represent significant difference between the negative and positive control. ***p < 0.001 represents significant difference between LPS-stimulation and co-treatment of LPS and Ak-N’m.

The effect of Ak-N’m on Mitogen-activated protein kinase (MAPK) signaling, which is downstream of the MyD88-dependent pathway, was targeted since the stimulation of LPS rapidly activates extracellular signal-regulated kinase (ERK), c-Jun N-terminal kinase (JNK), and p38. The activation of MAPK in macrophages was measured by Western blot analysis after 30 min stimulation with 1 μg/mL LPS. The phosphorylation of ERK and JNK was significantly inhibited by Ak-N’m treatment (2 μM) compared to the LPS-treated sample as the positive control (**Figure 8A**). Also, the protein expression and activation of nuclear factor kappa-light-chain-enhancer of activated B cells (NF-κB), a transcription factor that regulates immune response, including cytokine production, was examined. The peptide substantially suppressed both expression and activation of NF-κB protein (**Figure 8B**). To confirm this, nuclear translocation of NF-κB was examined by immunostaining. The reduced intensity of red fluorescence indicated the inhibition of NF-κB phosphorylation, leading to suppressed translocation shown by the merged signal of pink fluorescence (**Figure 8C**). Conclusively, the anti-inflammatory property of Ak-N’m was contributed by inhibition of MAPK signaling pathway, explaining the down-regulation of mRNA and protein levels of pro-inflammatory cytokines by the peptide.

### The Attenuation of the TRIF-Dependent Pathway Upon Ak-N’m Treatment

**Figure 9:**
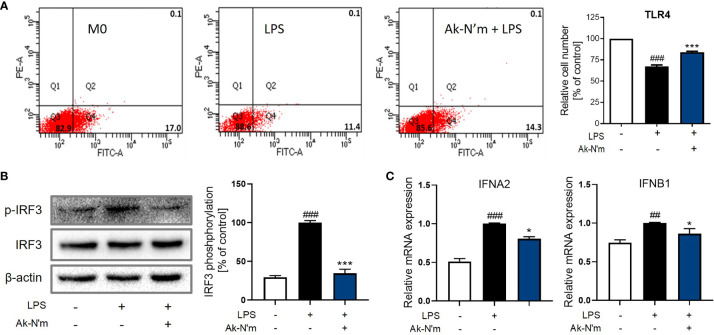
Ak-N’m treatment attenuated TRIF-dependent pathway. Ak-N’m inhibited the TRIF-dependent pathway in LPS-stimulated macrophages. **(A)** The endocytosis of TLR4 was attenuated *via* Ak-N’m treatment, where the decrease of surface TLR4 by LPS stimulation was significantly attenuated by the co-treatment of the peptide. **(B)** Transcription factor IRF3 was inactivated upon treatment of the peptide. **(C)** AkN’m suppressed the expression of Type 1 interferon genes IFNA2 and IFNB1. Data are presented as mean ± SEM. ^##^p < 0.01 and ^###^p < 0.001 represent significant difference between the negative and positive control. *p < 0.05 and ***p < 0.001 represent significant difference between LPS-stimulation and co-treatment of LPS and Ak-N’m.

Upon stimulation of LPS, TLR4 forms a heterodimer leading to the endocytosis that activates the TRIF-dependent pathway. Consequent phosphorylation of transcription factor interferon regulatory factor 3 (IRF3) leads to the production of pro-inflammatory molecules, including Type 1 interferon (IFN). The effect of Ak-N’m on the surface TLR4 was studied by flow cytometry. LPS-stimulated M0 macrophages with or without the presence of Ak-N’m were stained with anti-TLR4 antibodies for the detection of surface receptors. While 17% of M0 macrophages were detected with the staining of TLR4, the percentage of the detected cells decreased after the LPS stimulation. The co-treatment of LPS and Ak-N’m attenuated the decrease of the surface TLR4 up to 14.3% (**Figure 9A**). The results indicated that Ak-N’m was able to hinder the TLR4 endocytosis of the macrophage caused by LPS, suggesting the overall inhibition of the TRIF-dependent inflammation pathway.

Also, LPS-induced activation of IRF3 was significantly inhibited by the peptide, which was comparable to the negative control (**Figure 9B**). Investigation on the gene expression corroborated the inhibition of the TRIF-dependent pathway by Ak-N’m, where interferon alpha-2 (IFNA2) and beta-1 (IFNB1) were both down-regulated in the presence of the peptide (**Figure 9C**). In conclusion, novel functional peptides were rationally designed by implementing a series of *in silico* analysis, and Ak-N’m exhibited significant antibacterial and anti-inflammatory properties with reduced cytotoxicity. The mechanistic study of Ak-N’m revealed a significant effect on the bacterial membrane as well as TLR4 signaling in macrophages.

## Discussion

The global crisis of antibiotic-resistance bacteria has demanded the development of next-generation antimicrobial agents. Alongside with specific control of infection, there is an urgent need for discovering therapeutic agents for effective and systemic immune modulation. HDP is a crucial player in innate immunity, as it is expressed in various species exhibiting multifunctionalities. Studies on utilizing HDPs for therapeutic purposes are ardently being conducted because of their wide spectrum of activity, low cost, and high safety. In addition, technological advances in computation and *in silico* analysis have accelerated the new discovery and utilization of biological resources (29).

Previous studies on spiders focused on the phylogenic investigation (30), and the identification and characterization of bioactive molecules from the spiders have been conducted only on a few limited species. As the venom of the spider can be a great platform to excavate functional compounds, the transcriptome of the *A*. *koreana* spider was searched for a novel HDP. A transcript c32159 showed homology with the known spider toxin peptides and was predicted of its functional potency.

Based on the high potential of antimicrobial activity of the template sequence c32159, the functional enhancement on the anti-inflammatory effect of the peptide as well as the reduction of cytotoxicity was investigated by implementing *in silico* methods. Artificial intelligence technology is becoming a powerful tool that can overcome the traditional analytical methods, especially when it comes to vast data from heterogeneous origins (31). Here, multiple machine learning-based web tools were employed to predict biological activities as selected algorithms successfully extracted the characteristics of functional properties underlying structural and sequence data. The optimal 20-mer analog of c32159, which showed the highest dual-functionality of antimicrobial and anti-inflammatory properties as well as the lowest hemolytic activity, was selected based on the overall prediction scoring. Moreover, the selected sequence was subjected to amino acid mutation for each residue to enhance biological activity according to its original purpose. During the evaluation, computation of physiochemical properties and secondary structure modeling was performed, accommodating the comprehensive workflow for designing functional peptides. As a result, the N-terminus of c32159 and its modified sequence were presented and named Ak-N’ Ak-N’, respectively.

A series of experiments were conducted to validate the prediction results and the functional properties of the designed peptides. Both peptides exhibited antimicrobial activities against gram-positive and negative strains, including antibiotic-resistant bacteria, MRSA. The further mechanistic evaluation showed that peptides are capable of rapid bacterial membrane disruption, leading to the effective killing of the microbes. The characterization of peptides indicated the correlation between the structural features and function; As Ak-N’ and Ak-N’m were cationic helical peptides with a high net charge, it is feasible for the peptides to actively interact with bacterial biomembranes that are negatively charged by the presence of LPS and peptidoglycan. Cationic short peptides can easily accumulate onto bacterial membranes that lead to various phenomena, such as pore formation, membrane disruption, penetration, and interference of cellular components. (32, 33). In addition, the diversity of bacterial membrane components contributes to the selectivity of the peptides, showing specific and strong bactericidal effects against specific strains.

A major concern in the therapeutic use of peptides is inherent toxicity towards mammalian cells. The positively charged residues of cationic peptides often contribute to the hydrophobic interaction with RBC and eukaryotic cell membranes (34). The investigation of the cytotoxic effect of the peptides revealed that the modified sequence of Ak-N’m exhibited reduced toxicity of peptide Ak-N’m compared with Ak-N’ in both cell viability and hemolysis assays. Also, the stability of the peptides in human serum showed that the peptides were suitable for *in vitro* and future *in vivo* analysis.

HDPs have immunomodulatory effects along with various biological roles, including wound healing, angiogenesis, and cell differentiation. These peptides monitor the physiological environment of the host to maintain homeostasis and respond to various internal as well as external signals (35–38). Upon infection, stimulation by LPS leads to TLR4 activation of the macrophages that leads to a series of immune responses. We tested the immunomodulatory effects of Ak-N’ and Ak-N’m on the THP-1-derived macrophage model with LPS stimulation. The expression of pro-inflammatory mediators, including TNF-α, IL-1β, and IL6, was suppressed upon co-stimulation of LPS and the peptide, demonstrating the suppressive effects of the peptides on the TLR4 pathway. Ak-N’m showed enhanced anti-inflammatory activity compared with Ak-N’ in consensus with the prediction results from machine learning tools. The following examination of protein expression confirmed the inhibitory effect of Ak-N’m on inflammation-associated enzymes, COX2 and NOS2, by Ak-N’m. As cytokines involved in acute and latent immune responses were down-regulated, the potency of Ak-N’m on inhibiting downstream of TLR4 signaling was further investigated (39–41). The rapid inflammation response by the MyD88-dependent pathway was inhibited as the MAPK proteins and transcription factor NF-κB were inactivated. Also, the TRIF-dependent pathway, which is initiated by intracellular localization of TLR4, was attenuated by Ak-N’m, where the endocytosis of the receptor caused by LPS and expression of type 1 interferons were inhibited (42, 43). The overall results suggested that Ak-N’m, a novel peptide designed from spider toxin, is a functional peptide with dual antimicrobial and anti-inflammatory activities which can modulate multiple pathways. The utilization of such multifunctional peptides can be beneficial as they can target pathogens and host immunity simultaneously. The utilization of such multifunctional peptides can be beneficial as they can target pathogens and host immunity simultaneously. The rapid clearance of bacteria and inhibition of pro-inflammatory mediators prevent secondary infection and excessive damage to the tissue by modulating the host’s microenvironment.

Optimizing, stabilizing, and enhancing the therapeutic index can broaden the opportunity for peptides to be developed as therapeutics (44–46). Clinical trials on peptide agents, as well as exploration of new candidates, are being conducted with much effort, giving substantial attention towards peptides derived from biological resources. As shown in this study, the modification of the sequence by truncation or residue substitution has improved functionalities while lowering cytotoxicity. *In silico* analysis, including computation and artificial intelligence-based prediction, can be actively employed to explore and repurpose new functional molecules, compensating for time-consuming and labor-extensive experimental approaches. In conclusion, the utilization of indigenous spider transcript has led to the discovery of a novel toxin peptide with both antibacterial and anti-inflammatory activity. The results demonstrated a rational and successful design of novel functional peptides *via* the suggested workflow, offering a perspective on the efficient utilization of animal-derived biological resources that can contribute to future drug development.

## Data Availability Statement

The datasets presented in this study can be found in online repositories. The names of the repository/repositories and accession number(s) can be found below: NCBI GEO database accession number GSE197102.

## Author Contributions

MKS designed the study, conducted the experiments, acquired data, and wrote the manuscript. BL analyzed data and edited the manuscript. STK provided the specimen. JSY conducted experiments and revised the manuscript. J-SS reviewed the data and supervised the study. All authors contributed to the article and approved the submitted version.

## Funding

This work was supported by a grant from the National Institute of Biological Resources (NIBR), funded by the Ministry of Environment (MOE) of the Republic of Korea (NIBR202134205).

## Conflict of Interest

The authors declare that the research was conducted in the absence of any commercial or financial relationships that could be construed as a potential conflict of interest.

## Publisher’s Note

All claims expressed in this article are solely those of the authors and do not necessarily represent those of their affiliated organizations, or those of the publisher, the editors and the reviewers. Any product that may be evaluated in this article, or claim that may be made by its manufacturer, is not guaranteed or endorsed by the publisher.
