# Supplementary material for: Designing a Novel Functional Peptide With Dual Antimicrobial and Anti-inflammatory Activities via in Silico Methods
Source: Front Immunol. 2022 Apr 1;13:821070. doi: 10.3389/fimmu.2022.821070 (PMC9010562; doi:10.3389/fimmu.2022.821070)
Supplement: Supplementary file 1 [file DataSheet_1.docx]

Supplementary Material

**Supplementary Table 1. Primer sets used in the study for qRT-PCR.**

| Gene | Sequence | |
| --- | --- | --- |
| GAPDH | F | TAT GAC AAC AGC CTC AAG AT |
|  | R | GAG TCC TTC CAC GAT ACC |
| TNF-α | F | AGA GAA GCA ACT ACA GAC C |
|  | R | CAG TAT GTG AGA GGA AGA GAA |
| IL-1β | F | GGC TTA TTA CAG TGG CAA TG |
|  | R | TAG TGG TGG TCG GAG ATT |
| IL-6 | F | ACC TCA GAT TGT TGT TGT T |
|  | R | AGT GTC CTA ACG CTC ATA |
| IFNA2 | F | TGG GCT GTG ATC TGC CTC AAA C |
|  | R | CAG CCT TTT GGA ACT GGT TGC C |
| IFNB1 | F | CTT GGA TTC CTA CAA AGA AGC AGC |
|  | R | TCC TCC TTC TGG AAC TGC TGC A |

**Supplementary Table 2. Functional prediction screening of c352159 using 20-mer window.**

| Sequence (20-mer) | AIP prediction | | | AMP prediction | | | | Hemolysis prediction | | |
| --- | --- | --- | --- | --- | --- | --- | --- | --- | --- | --- |
|  | **AIPpred** | **PreAIP** | **AntiInflam** | **ADAM** | **AmpGram** | **CAMPR3 (SVM)** | **DBAASP** | **DBAASP**  **(erythrocyte)** | **HAPPEN** | **HemoPI** |
| FKGLAKLLKIGLKALAKVIQ | 0.5558 | 0.618 | 0.893109 | 2.56 | 1 | 0.960 | AMP | Active | 0.489 | 0.50 |
| KGLAKLLKIGLKALAKVIQK | 0.5581 | 0.648 | 0.871363 | 2.76 | 1 | 0.780 | AMP | Active | 0.560 | 0.48 |
| GLAKLLKIGLKALAKVIQKA | 0.5442 | 0.654 | 0.699771 | 2.51 | 1 | 0.987 | AMP | Active | 0.798 | 0.56 |
| LAKLLKIGLKALAKVIQKAL | 0.5558 | 0.657 | 0.549693 | 2.38 | 1 | 0.960 | AMP | Active | 0.696 | 0.59 |
| AKLLKIGLKALAKVIQKALP | 0.5674 | 0.648 | 0.679345 | 2.21 | 1 | 0.952 | AMP | Active | 0.309 | 0.52 |
| KLLKIGLKALAKVIQKALPK | 0.6209 | 0.66 | 0.77243 | 2.46 | 1 | 0.860 | AMP | Active | 0.599 | 0.44 |
| LLKIGLKALAKVIQKALPKA | 0.6279 | 0.627 | 0.608574 | 2.21 | 1 | 0.989 | AMP | Active | 0.798 | 0.52 |
| LKIGLKALAKVIQKALPKAA | 0.5860 | 0.643 | 0.34119 | 2.3 | 1 | 0.963 | AMP | Active | 0.246 | 0.49 |
| KIGLKALAKVIQKALPKAAK | 0.5186 | 0.557 | 0.114628 | 2.61 | 1 | 0.832 | AMP | Active | 0.152 | 0.38 |
| IGLKALAKVIQKALPKAAKA | 0.5209 | 0.577 | 0.165459 | 2.53 | 1 | 0.994 | AMP | Active | 0.523 | 0.45 |
| GLKALAKVIQKALPKAAKAG | 0.5186 | 0.55 | 0.118174 | 2.21 | 1 | 0.976 | AMP | Active | 0.243 | 0.42 |
| LKALAKVIQKALPKAAKAGK | 0.4953 | 0.572 | -0.04695 | 2.31 | 1 | 0.929 | AMP | Active | 0.020 | 0.45 |
| KALAKVIQKALPKAAKAGKA | 0.4860 | 0.503 | -1.19165 | 2.49 | 1 | 0.826 | AMP | Active | 0.047 | 0.48 |
| ALAKVIQKALPKAAKAGKAL | 0.4837 | 0.517 | -1.19165 | 2.25 | 1 | 0.976 | AMP | Active | 0.141 | 0.49 |
| LAKVIQKALPKAAKAGKALA | 0.4884 | 0.514 | -1.19165 | 2.25 | 1 | 0.968 | AMP | Active | 0.015 | 0.49 |
| AKVIQKALPKAAKAGKALAK | 0.4837 | 0.53 | -1.19165 | 2.49 | 1 | 0.923 | AMP | Active | 0.003 | 0.48 |
| KVIQKALPKAAKAGKALAKS | 0.4860 | 0.537 | -1.20816 | 2.18 | 1 | 0.808 | AMP | Active | 0.017 | 0.45 |
| VIQKALPKAAKAGKALAKSM | 0.4907 | 0.53 | -1.30562 | 1.42 | 1 | 0.982 | AMP | Active | 0.014 | 0.43 |
| IQKALPKAAKAGKALAKSMA | 0.5209 | 0.547 | -1.32062 | 1.74 | 1 | 0.961 | AMP | Active | 0.005 | 0.45 |
| QKALPKAAKAGKALAKSMAD | 0.5302 | 0.549 | -1.38918 | 0.98 | 1 | 0.314 | AMP | Not Active | 0.004 | 0.48 |
| KALPKAAKAGKALAKSMADE | 0.4977 | 0.564 | -1.39325 | 1.12 | 0.989 | 0.216 | AMP | Not Active | 0.012 | 0.48 |
| ALPKAAKAGKALAKSMADEN | 0.4860 | 0.537 | -1.52363 | 1.47 | 0.9447 | 0.373 | AMP | Not Active | 0.005 | 0.48 |
| LPKAAKAGKALAKSMADENA | 0.5000 | 0.513 | -1.54466 | 1.47 | 0.7745 | 0.295 | Non-AMP | Not Active | 0.002 | 0.48 |
| PKAAKAGKALAKSMADENAI | 0.5070 | 0.504 | -1.59413 | 1.61 | 0.7516 | 0.355 | Non-AMP | Not Active | 0.008 | 0.46 |
| KAAKAGKALAKSMADENAIR | 0.5140 | 0.503 | -0.53821 | 1.41 | 0.6683 | 0.362 | Non-AMP | Not Active | 0.001 | 0.46 |
| AAKAGKALAKSMADENAIRQ | 0.5395 | 0.501 | -0.37937 | 1 | 0.6293 | 0.640 | Non-AMP | Not Active | 0.001 | 0.46 |
| AKAGKALAKSMADENAIRQQ | 0.5442 | 0.496 | -0.16625 | 0.42 | 0.5318 | 0.454 | Non-AMP | Not Active | 0.001 | 0.46 |
| KAGKALAKSMADENAIRQQN | 0.6070 | 0.485 | -0.12474 | 0.78 | 0.4081 | 0.277 | Non-AMP | Not Active | 0.004 | 0.46 |
| AGKALAKSMADENAIRQQNQ | 0.6442 | 0.476 | -0.13316 | 0.52 | 0.2861 | 0.425 | Non-AMP | Not Active | 0.002 | 0.47 |

**Supplementary Table 3. Physiochemical properties of selected five candidates.**

| Sequence | FKGLAKLLKI  GLKALAKVIQ | KGLAKLLKIG  LKALAKVIQK | AKLLKIGLKA  LAKVIQKALP | KLLKIGLKAL  AKVIQKALPK | LKIGLKALAK  VIQKALPKAA |
| --- | --- | --- | --- | --- | --- |
| Net charge | +5 | +6 | +5 | +6 | +5 |
| Mw | 2152.75 | 2133.75 | 2116.72 | 2173.81 | 2074.64 |
| pI | 11.28 | 11.37 | 11.37 | 11.37 | 11.28 |
| Solubility | Good | Good | Good | Good | Good |
| Hydrophobic face | IGALVGLLLFI | IGALVGLLLILAA | ALVGALLILAA | ALVGALLILLA | VGALLILLA |
| Helical structure | 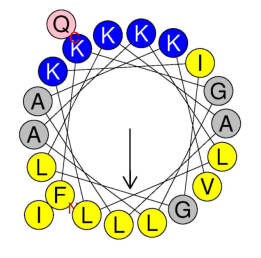 | 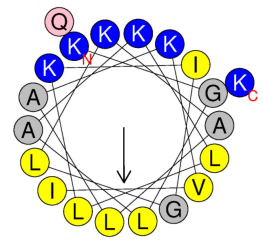 | 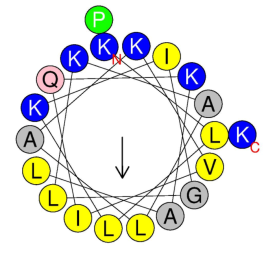 | 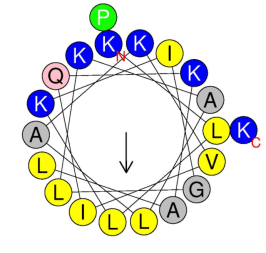 | 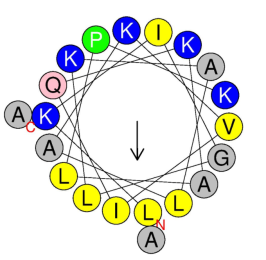 |


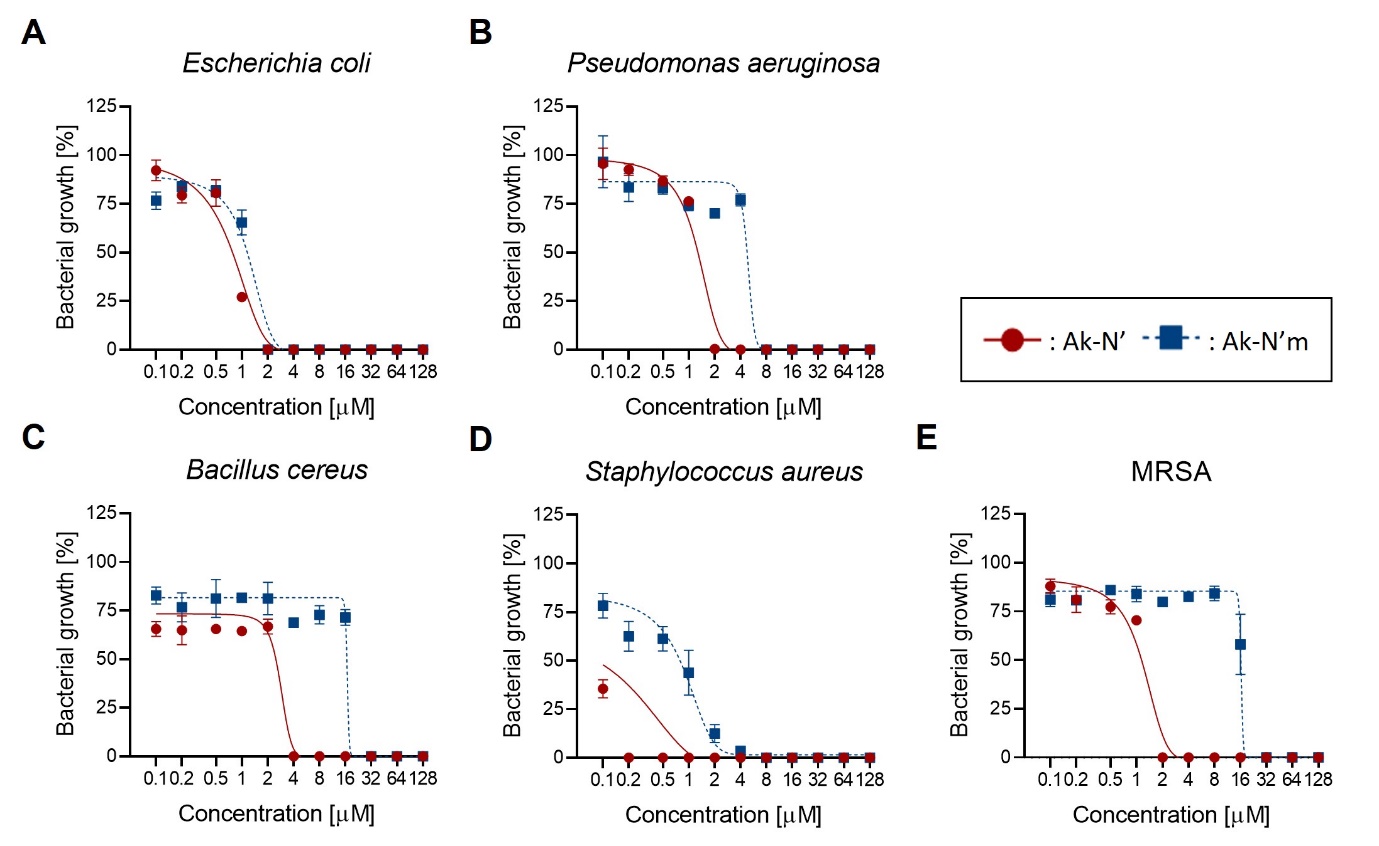


**Supplementary Figure 1**. **Bacterial growth inhibition by Ak-N’ and Ak-N’m.**

Growth inhibition of Ak-N’ and Ak-N’m was investigated by the microdilution method. Each bacterial strain was incubated with an equal volume of peptides to reach the final concentration ranging 0.1~128 μM for 24 h. Relative bacterial growth of **(A)** *E*. *coli*, **(B)** *P. aeruginosa*, **(C)** *B*. *cereus*, **(D)** *S*. *aureus*, and **(E)** MRSA was shown.


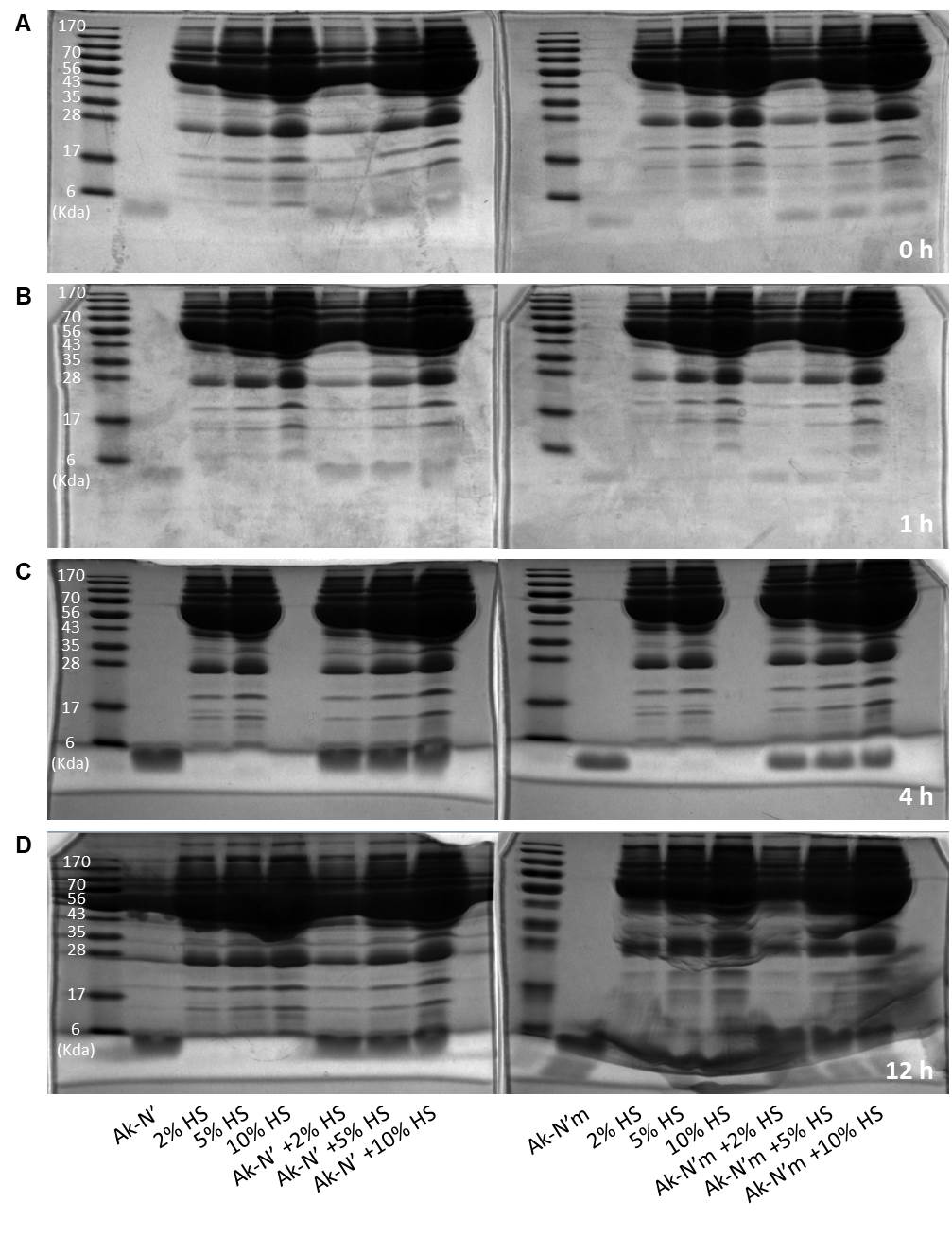


**Supplementary Figure 2**. **Stability of Ak-N’ and Ak-N’m in human serum.**

Peptide stability in HS was studied using SDS-PAGE followed by coomassie blue staining. 25 μg of Ak-N’ and Ak-N’m were incubated with 2%, 5%, 10% human serum at 37 °C for **(A)** 0h, **(B)** 1h, **(C)** 4h, and **(D)** 12h. Both peptides were observed by staining after 12h incubation with HS.
